# *VEGF-A* and *ICAM-1* Gene Polymorphisms as Predictors of Clinical Outcome to First-Line Bevacizumab-Based Treatment in Metastatic Colorectal Cancer

**DOI:** 10.3390/ijms20225791

**Published:** 2019-11-18

**Authors:** Apostolos Papachristos, Polychronis Kemos, Theodora Katsila, Eirini Panoilia, George P. Patrinos, Haralabos Kalofonos, Gregory B. Sivolapenko

**Affiliations:** 1 Laboratory of Pharmacokinetics, Department of Pharmacy, School of Health Sciences, University of Patras, Patra 26504, Greece; alkispapachristos@gmail.com (A.P.); irenepanoelia@yahoo.gr (E.P.); 2 Division of Cancer, University College London Hospital NHS Foundation Trust, London NW12BU, UK; 3 Centre for Immunobiology, Blizard institute, Queen Mary University of London, London E12AT, UK; p.kemos@qmul.ac.uk; 4 Laboratory of Pharmacogenomics and Individualized Therapy, Department of Pharmacy, School of Health Sciences, University of Patras, Patra 26504, Greece; thkatsila@eie.gr (T.K.); gpatrinos@upatras.gr (G.P.P.); 5 Institute of Chemical Biology, National Hellenic Research Centre, Athens 11635, Greece; 6 Division of Medical Oncology, University Hospital of Patras, Patra 26504, Greece; hkalofon@yahoo.gr

**Keywords:** pharmacogenomics, anti-angiogenetic therapy, colorectal cancer, survival, bevacizumab, predictive biomarkers, VEGF-A, ICAM-1, BRAF

## Abstract

Bevacizumab is used to treat metastatic colorectal cancer (mCRC). However, there are still no available predictors of clinical outcomes. We investigated selected single nucleotide polymorphisms (SNPs) in the genes involved in VEGF-dependent and -independent angiogenesis pathways and other major intracellular signaling pathways involved in the pathogenesis of mCRC as an attempt to find predictors of clinical outcome. Forty-six patients treated with first-line bevacizumab-based chemotherapy were included in this study with a 5 year follow up. Genomic DNA was isolated from whole blood for the analysis of *VEGF-A* (rs2010963, 1570360, rs699947), *ICAM-1* (rs5498, rs1799969) SNPs and from tumor tissue for the detection of genomic variants in *KRAS*, *NRAS*, *BRAF* genes. PCR and next generation sequencing were used for the analysis. The endpoints of the study were progression-free survival (PFS) and overall survival (OS). The *VEGF-A* rs699947 A/A allele was associated with increased PFS (*p* = 0.006) and OS (*p* = 0.043). The *ICAM-1* rs1799969 G/A allele was associated with prolonged OS (*p* = 0.036). Finally, *BRAF* wild type was associated with increased OS (*p* = 0.027). We identified *VEGF-A* and *ICAM-1* variants in angiogenesis and other major intracellular signaling pathways, such as *BRAF*, that can predict clinical outcome upon bevacizumab administration. These identified biomarkers could be used to select patients with mCRC who may achieve long-term responses and benefit from bevacizumab-based therapies.

## 1. Introduction

In 1971, Judah Folkman first described angiogenesis and its contribution to tumor growth [1]. Since then, the angiogenesis pathway has been extensively studied. The dominant factor controlling angiogenesis is the vascular endothelial growth factor (VEGF) glycoprotein [2]. The VEGF family comprises five members (VEGFA-E), and placental growth factor (PlGF). VEGFs bind with high affinity to receptors (VEGFR-1 and VEGFR-2) and promote angiogenic signals to vascular endothelium [3]. VEGF-A is considered the major activator of angiogenesis. It acts selectively on vascular endothelial cells, stimulating both normal and abnormal angiogenesis [4]. VEGF-A binds to VEGFR-1 and VEGFR-2, although the VEGF-A/VEGFR-2 pathway is considered the major activator of angiogenesis [5]. Another molecule involved in angiogenesis is ICAM-1 (also known as CD54), a member of the immunoglobulin supergene family. It is constitutively localized on the cell surface and is abundantly expressed in a variety of cell types, including fibroblasts, leukocytes, keratinocytes, and endothelial cells. ICAM-1 contains five extracellular immunoglobulin-like domains that function in adhesive cell–cell or cell–matrix interactions by binding to two integrins belonging to the β2 subfamily: lymphocyte function-associated antigen-1 (LFA-1; also known as CD11α/CD18) and macrophage antigen-1 (Mac-1; also known as CD11b/CD18) [6]. Importantly, ICAM-1 is markedly expressed in many different types of human cancer cells, including lung, pancreatic, breast, and prostate cancer cells, as well as in glioma [7].

Several studies have reported the prognostic and predictive significance of VEGF and VEGFR in colorectal [8,9], lung [10], gastric [11] and pancreatic [12] cancers. Interestingly, in vivo experiments in mice have also shown that highly expressed ICAM-1 may mediate resistance to antiangiogenic therapy [13]. Angiogenesis and especially, VEGF-A have become attractive pharmacologic targets in colorectal cancer (CRC), which is the third most common cancer in males and the second in females; only in 2015, 1.65 million new CRC cases and almost 835,000 deaths were recorded [14]. The pathogenesis of CRC also involves the accumulation of genetic and epigenetic modifications within the pathways that regulate proliferation, apoptosis and angiogenesis. *RAS* and *BRAF* genomic variants are of prognostic and predictive value in metastatic colorectal cancer (mCRC). *KRAS* genomic variants involving either codon 12 or 13 can be identified in 12–75% CRCs and they have been independently associated with a worse prognosis. *NRAS* mutations are also associated with an inferior prognosis. Similarly, *BRAF* activating mutations that occur mostly in codon 600 (V600E) are present in less than 10% tumors and represent a strong negative prognostic marker [15,16].

Bevacizumab, a recombinant humanised IgG1 monoclonal antibody, is the first agent approved against VEGF-A. Bevacizumab can be used as monotherapy or in combination with chemotherapy for the treatment of mCRC, metastatic breast cancer, unresectable advanced, metastatic or recurrent non-small cell lung cancer, advanced or metastatic renal cell cancer, advanced ovarian and cervical cancers and as monotherapy for advanced, recurrent glioblastoma [17]. The mechanism of action of bevacizumab includes binding to circulating VEGF-A and blocking of VEGF-A binding to its receptors (VEGFR-1 and VEGFR-2) on the surface of endothelial cells, which results in the inhibition of tumor angiogenesis, growth, and metastases [18].

The research for biomarkers of angiogenesis and anti-angiogenesis and their successful use in the development of angiogenesis inhibition therapy is an ongoing challenge. However, no validated biomarkers are currently available to guide patient selection for treatment with bevacizumab. In the present study, we investigated the role of selected single nucleotide polymorphisms (SNPs) in the *VEGF-A* and intracellular adhesion molecule-1 (*ICAM-1*) angiogenesis genes, as well as in the *KRAS*, *NRAS* and *BRAF* genes, in order to predict clinical outcome in mCRC patients treated with first-line bevacizumab in combination with chemotherapy (fluoropyrimidines and oxaliplatin or irinotecan).

## 2. Results 

A total of 46 consecutive patients with mCRC were enrolled in the study. Overall, patients had a mean age of 64.5 years (range from 31 to 86) and were predominantly male (28/46; 61%) (Table 1). The most commonly used initial treatment was BEV-FOLFOX (46%) and the most common maintenance treatment was bevacizumab monotherapy (30%) (Table 2). The mean number of metastatic sites was 2 (range from 1 to 5). Most patients had wild-type variants of *VEGF-A* SNPs: rs2010963 (58.7%), rs1570360 (67.4%) and rs699947 (74%). Similarly, the wild-type *ICAM-1* gene rs1799969 was dominant (78.3%), but 50% of patients were heterozygous for rs5498. Fifty percent of patients had *KRAS* wild-type tumors and 61.2% presented with *NRAS* mutations. The vast majority (81.8%) of patients had wild-type *BRAF* tumors. Genotype frequencies are summarised in Table 1.

After a median follow up of 60 months, 88.1% of patients progressed and 58% died. For all patients, median progression-free survival (PFS) and overall survival (OS) were 9 and 18 months, respectively. In terms of the best response, complete response (CR) was achieved by one patient, partial response (PR) by 16 (34.8%) patients, stable disease by 25 (54.4%) patients, and progressive disease (PD) in 4 (8.7%) patients.

**Figure 1:**
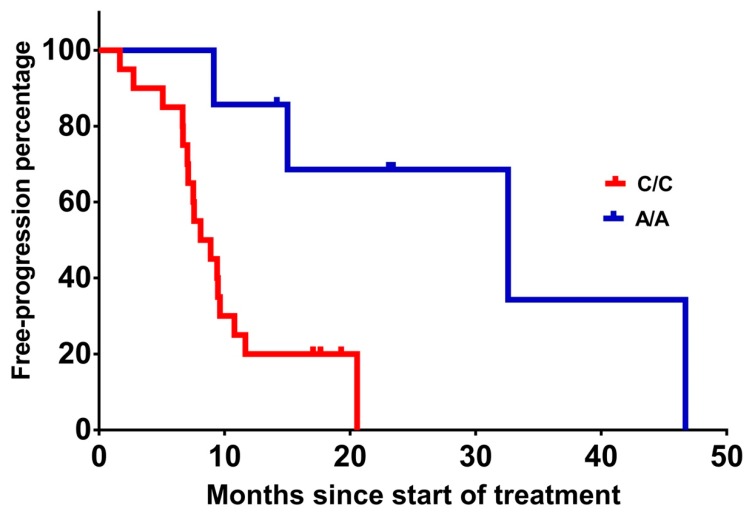
Progression-free survival (PFS) from the treatment initiation of patients with *VEGF-A* rs699947 A/A vs. C/C (31.1 vs. 10.1 months, *p* = 0.006).

When progression-free survival was measured, patients homozygous for *VEGF-A* rs699947 A/A had significantly (*p* = 0.006) prolonged PFS of 32.6 months compared with 8.1 months for the carriers of the wild-type C/C variant (Table 3 and Figure 1). No other statistically significant associations were found between *VEGF-A* rs1570360 and rs2010963, *ICAM-1* rs5498 and rs1799969, *KRAS*, *NRAS*, and *BRAF* gene variants and PFS (Table 3). Chemotherapy type (irinotecan-based vs. oxaliplatin-based) was not associated with PFS.

**Figure 2:**
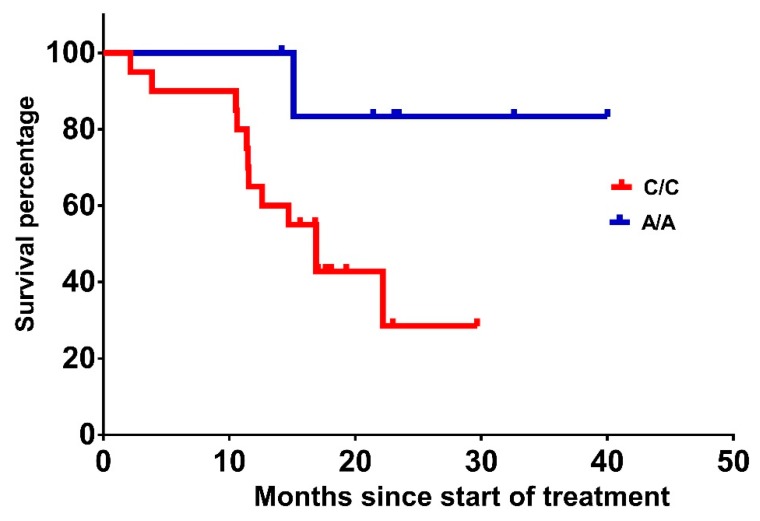
OS from treatment initiation of patients with *VEGF-A* rs699947 A/A vs. C/C (52 vs. 18.1 months, *p* = 0.043).

OS was also significantly (*p* = 0.043) prolonged to 59.4 months in carriers of *VEGF-A* rs699947 A/A compared with 16.9 months for carriers of the wild-type C/C variant (Table 3 and Figure 2). Patients heterozygous for *VEGF-A* rs1570360 G/A had longer OS compared with carriers of the G/G or A/A variants (43.2, 27.8 and 39.2 months, respectively), albeit that the difference did not meet statistical significance (*p* = 0.074). None of the variants of the *VEGF-A* rs2010963 were associated with OS (*p* = 0.811).

**Figure 3:**
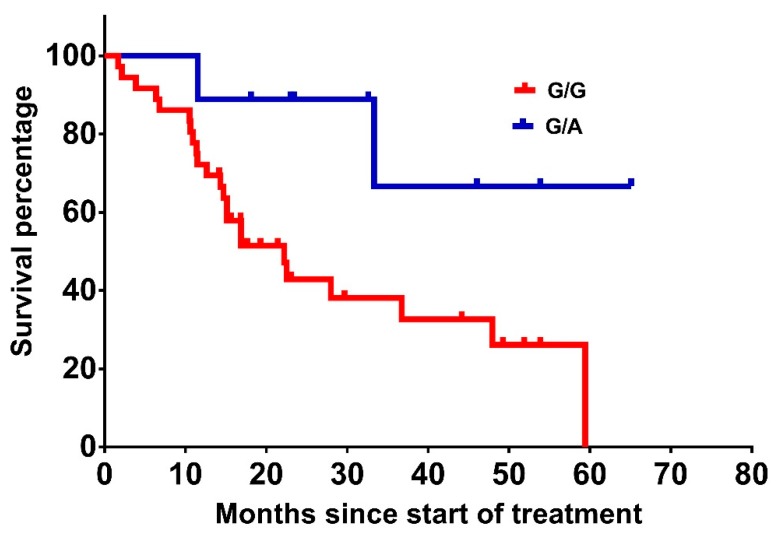
OS from treatment initiation of patients with *ICAM-1* rs1799969 G/A vs. G/G (48.7 vs. 29.1 months, *p* = 0.036).

Interestingly, patients harbouring the *ICAM-1* allele rs1799969 G/A attained a median OS of 48.7 months compared to 29.1 months in patients harbouring the G/G allele (*p* = 0.036) (Table 3 and Figure 3). In contrast, *ICAM-1* rs5498 was not significantly associated with OS (*p* = 0.159).

The median OS of patients with wild-type *BRAF* was significantly longer compared with patients with the mutant *BRAF* (16.7 vs. 6.8 months, *p* = 0.027; Table 3). *KRAS* and *NRAS* were not significantly associated with OS (*p*-values 0.511 and 0.374, respectively; Table 3). Chemotherapy type (irinotecan-based vs. oxaliplatin-based) was not associated with OS.

## 3. Discussion

Although bevacizumab is widely used in oncology, contrary to other therapeutic classes, there remains a lack of validating predictive factors for treatment outcomes [19]. In recent years, the research for factors predictive of anti-VEGF treatment outcomes and especially for bevacizumab response has attracted great scientific interest. Several clinical studies in patients with mCRC have noted a relationship between the concentration of symptoms such as hypertension, biomarkers (e.g., lactate dehydrogenase, ICAM, E-selectin, endothelial nitric oxide synthase) and the SNPs of genes involved in the angiogenesis pathway and the response to bevacizumab [20,21,22,23,24,25,26]. It is not clear whether the genetic variants associated with clinical outcomes are due to lower angiogenesis activity or to lower in vivo affinity between bevacizumab and VEGF, as indicated in previous study [27].

Our results demonstrate the significant association between the specific polymorphisms in VEGF-dependent and non-VEGF-dependent genes and the improved clinical outcomes in patients with mCRC receiving bevacizumab in combination with chemotherapy as first-line treatment. 

Patients homozygous for *VEGF-A* rs699947 A/A had statistically significantly prolonged PFS and OS as compared to carriers of the wild-type C/C variant (PFS: 32.6 vs. 8.1 months and OS: 59.4 vs. 16.9 months, respectively). In addition, increasing OS trends were noticed in carriers of *VEGF-A* rs1570360 G/A and A/A of 43.2 months and 39.2 months, respectively, vs. 27.8 months of the corresponding G/G variant. The correlation between certain *VEGF-A* variants and the outcome of bevacizumab treatment has been previously explored, and our results are aligned with these studies. Genotyping in 173 mCRC patients treated with bevacizumab in combination with FOLFIRI or CapIRI showed that rs1570360 G/G was significantly associated with inferior OS compared to G/A [28]. Furthermore, a phase III study of patients with metastatic breast cancer receiving bevacizumab and paclitaxel showed that the median OS was significantly longer for carriers of *VEGF-A* rs699947 A/A and rs1570360 A/A [29]. The correlation between *VEGF-A* SNPs and treatment efficacy was also examined in patients receiving bevacizumab and sorafenib for recurrent glioblastoma; mutant *VEGF-A* allele rs699947 was associated with a 6 month increase in PFS whilst mutant *VEGF-A* rs1570360 was associated with a 6 month decrease in PFS [30]. Nonetheless, there are reports where no correlation could be identified between *VEGF-A* polymorphisms and PFS or OS in patients with cancer and treated with bevacizumab. Ulivi et al. [24] and Etienne-Grimaldi et al. [31] tested the impact of *VEGF-A* gene polymorphisms (including rs2010963, rs1570360, rs699947) in patients receiving bevacizumab for mCRC and metastatic breast cancer. No correlation was reported between any SNP of *VEGF-A* with response rate, PFS or OS. The disparity between our results and those of Ulivi et al. may be due to the different study designs and/or the longer follow-up period in the present study (median of 60 vs. 36 months, respectively).

In our study, OS was favourably associated with the *ICAM-1* rs1799969 G/A variant as compared with the G/G variant (48.7 vs 29.1 months, respectively; *p* = 0.036). Our results show that mCRC carriers of the *ICAM-1* rs1799969 G/A allele have a benefit of 19.6 months of survival compared to carriers of the wild-type G/G allele when treated with bevacizumab. To our knowledge, this is the first time that a clear, significant association has been observed between the *ICAM-1* gene and OS in cancer patients treated with bevacizumab. There are reports in the literature which describe the association between ICAM-1 blood concentrations and clinical outcomes. In 99 mCRC patients treated with first-line bevacizumab in combination with mFOLFOX-6 or XELOX, high plasma ICAM blood levels (>190.0 ng/mL) were strongly associated with shorter OS (*p* = 0.003) [32]. Furthermore, in a phase II/III study (E4599) where patients with advanced non-small cell lung cancer were randomised to receive carboplatin and paclitaxel with or without bevacizumab, it was found that regardless of treatment arm, low ICAM-1 levels (≤260.5 ng/mL) were prognostic for survival and predictive of response to treatment (32% vs. 14%, *p* = 0.02) and 1 year survival (65% vs. 25%). [21]. In addition, a trend toward a higher risk of death and high ICAM-1 levels (*p* = 0.06) was revealed by correlative studies of a phase II trial assessing the efficacy and safety of a bevacizumab and cisplatin, etoposide combination in 63 patients with extensive stage small-cell lung cancer [33]. Nonetheless, in the ABIGAIL study [34], baseline and dynamic changes in plasma levels of ICAM-1 did not correlate with response to bevacizumab in 303 chemotherapy-naïve patients with non-small cell lung cancer who were treated with bevacizumab in combination with chemotherapy (carboplatin and gemcitabine or carboplatin and paclitaxel).

Several clinical studies have demonstrated a strong association between *BRAF* mutations and inferior survival in patients with CRC [35]. We also confirmed that *BRAF* mutation status was an important predictor for clinical outcomes, as patients with wild type *BRAF* had a significant survival benefit of 9.9 months. In line with our results, Bruera et al. [36] reported an increased OS of 28 months in wild-type *BRAF* tumors compared to 11 months in patients with mutant-*BRAF* tumors. Tol et al. [37] reported that the *BRAF* mutation is a negative prognostic marker for patients receiving treatment with bevacizumab and CapOX; OS was significantly longer in patients with wild-type *BRAF* compared with patients with mutant *BRAF* (24.6 and 15 months, respectively). Ince et al. reported similar results in patients treated with bevacizumab and FOLFIRI, with OS of 26.3 and 15.9 months in wild type and mutant tumors, respectively, although their results did not reach statistical significance [38].

Our results show that *KRAS* and *NRAS* were not predictive, neither for PFS nor for OS in patients with mCRC who were treated with bevacizumab-containing first-line treatment. The poor survival and the prognostic impact of the *KRAS* mutation in CRC has been previously reported [39]. Prince et al. also reported that *KRAS* gene mutation status was neither prognostic nor predictive for PFS, OS and outcomes in patients with mCRC receiving capecitabine and bevacizumab [40]. Similar results were also presented by Hurwitz et al. [41], who reported that the clinical benefit of bevacizumab in mCRC was independent of *KRAS* mutation status.

In conclusion, we identified major angiogenesis and intracellular signaling pathway polymorphisms as predictors of response to first-line bevacizumab-based treatment in metastatic colorectal cancer. In a pragmatic and real-world study, we showed that certain genetic variants of VEGF-A, ICAM-1 and BRAF are associated with longer responses and higher benefit during treatment with bevacizumab. It would be reasonable to suggest that these biomarkers could be incorporated in decision-making processes when bevacizumab, whether reference or biosimilar, is administered in patients with mCRC, such as patient selection, randomization, stratification in clinical development programs, and optimal treatment selection during standard care and reimbursement. It cannot be excluded that the non-highlighted polymorphisms may also play a role in the recorded PFS and OS but with a smaller effect size or potentially in combination. In the future, moving from conventional treatment to precision medicine, the verification and validation of existing SNPs as well as the identification of newer ones is anticipated to greatly impact on the management of not only mCRC but also patients with other types of cancer.

## 4. Materials and Methods

### 4.1. Design

In total, 46 patients with histologically confirmed mCRC who started first-line bevacizumab-based treatment between April 2013 and April 2014 were studied. The data cut-off was July 2018. Patients were 18 years of age or older and had an ECOG performance status of 0–2. All patients received first-line treatment with bevacizumab (Avastin^®^, Roche, Basel, Switzerland) in combination with oxaliplatin or irinotecan and fluoropyrimidine-based chemotherapy in accordance with the bevacizumab Summary of Product Characteristics and standard care. Bevacizumab was administered as an intravenous infusion at a dose of 5 mg/kg once every 2 weeks in combination with 5-fluorouracil/leucovorin/irinotecan or oxaliplatin (BEV-FOLFIRI or BEV-FOLFOX, respectively) or at a dose of 7.5 mg/kg once every 3 weeks in combination with capecitabine/irinotecan or oxaliplatin (BEV-CapIRI or BEV-CapOX, respectively) in 3 week cycles. Treatment was initially administered for six (BEV-FOLFIRI, BEV-FOLFOX) or four (BEV-CapIRI, BEV-CapOX) cycles; patients who responded to treatment continued with bevacizumab-based maintenance treatment. Radiographic evaluation was performed every 8–12 weeks or when clinically indicated. The response was evaluated according to RECIST criteria version 1.1. The treating physicians decided the maintenance treatment. The endpoints of the study were progression-free survival (PFS), and overall survival (OS).

This study was conducted at the Department of Oncology, University Hospital of Patras, Greece in accordance with the Declaration of Helsinki and the International Conference on Harmonisation (ICH) Good Clinical Practice. Approval was obtained by the Hospital’s Ethics Committee (3221/7, 12.02.2013). Prior to study enrolment, all patients provided signed informed consent.

### 4.2. Pharmacogenetic Analyses

Whole-blood samples were collected at baseline in order to analyse the *VEGF-A* (rs2010963, rs1570360, rs699947) and *ICAM-1* (rs5498, rs1799969) SNPs. Blood samples were collected in serum separator tubes and could clot for 30 min. After centrifugation at 1000× *g* for 20 min, the serum was removed and stored in aliquots at ≤−20 °C until analysis. Genomic DNA was extracted from peripheral blood leukocytes from patients using the Gentra Puregene Blood kit (QIAGEN) [42]. DNA concentrations were determined by measuring the optical density at 260 nm with a UV–Vis spectrophotometer (NanoDrop 2000, Thermo Fisher Scientific, Waltham, MA, USA). DNA purity, which is indicated by the ratio of optical density at 260 and 280 nm, was 1.7–1.9. *VEGF-A* (rs699947, rs1570360, and rs2010963) and *ICAM-1* (rs1799969, rs5498) genomic variants were analysed by polymerase chain reaction (PCR) according to the KAPA2G Fast HotStart protocol (Kapa Biosystems, Wilmington, MA, USA); detailed information per genomic variant amplification conditions is available upon request. PCR products were separated on 1% w/v agarose gels stained with Midori Green and were purified using the PCR and DNA Fragment Purification kit (Dongsheng Biotech, Guangzhou, China, DNA purity 1.7–1.9). Following purification, samples were subjected to direct DNA sequence analysis on an ABI Prim 3130xl DNA Analyser (Applied Biosystems, Foster City, CA, USA) using the Big Dye^®^ Terminator v3.1 Cycle Sequencing Kit (Applied Biosystems), according to the manufacturer’s instructions.

*KRAS*, *NRAS* (exons 2, 3, 4) and *BRAF* (exons 11 and 15) mutation status was determined in genomic DNA extracted from formalin-fixed, paraffin-embedded tissue samples from the patients with a QIAmp DNA FFPE tissue kit (Qiagen/MagCore Genomic DNA FFPE One-Step Kit, RBCBioscience, New Taipei City, Taiwan). Mutations in exons 2, 3, 4 of the *KRAS* and *NRAS* genes, and in exons 11 and 15 of *BRAF* gene were detected with the method of targeted realignment (Ion AmpliSeqPanel, Thermo Fisher Scientific). Next generation sequencing platform Ion proton (Thermo Fisher Scientific) was used for sequencing. The method’s detection limit was 2–5% (mutant/wild-type alleles) [43,44].

### 4.3. Statistics and Data Analysis

All categorical data, including therapy methods and polymorphisms, were tabulated and presented as frequencies and counts. Statistical significance was set at *p* < 0.05. For the time-to-event analyses, we used the Kaplan–Meier estimate, and all lost to follow-up cases were censored up to the most recent available time-point. The OS and PFS median estimates are presented with 95% confidence intervals. The log-rank test was used for the comparison of the OS and PFS distributions for different polymorphisms, while the effects of age and sex were investigated with multiple analyses using Cox regression. In terms of the sample size calculation, there were feasibility limitations in recruitment and therefore, the investigators calculated the effect size that this study could capture for the pre-determined number of patients. At a power level of 80% and alpha set as 0.05, for 46 patients with 60 months of follow up and 1 year of recruitment, we can detect at least 1.5 years of difference in the median of PFS and OS between different polymorphisms. The Kaplan–Meier graphs were generated using GraphPad Prism version 7 for Windows, GraphPad Software, La Jolla, California, USA. The analyses were performed using SAS version 9.4, SAS Institute Inc. 2015. SAS/IML^®^ 14.1 User’s Guide. Cary, NC: SAS Institute Inc.

## Figures and Tables

**Table 1:** Baseline patient characteristics and genotype frequencies.

Characteristics	All Treated Patients (*n* = 46)
Sex, *n* (%)	
Female	18 (39%)
Male	28 (61%)
Age (years), *n* (%)	
<55	14 (30%)
55–65	12 (26%)
>65	20 (44%)
Metastatic site, *n* (%)	
1	15 (33%)
2	12 (26%)
>2	19 (41%)
VEGF rs2010963, *n* (%)	
G/G	27 (58.7%)
G/C	13 (28.3%)
C/C	6 (13%)
VEGF rs1570360, *n* (%)	
G/G	31 (67.4%)
G/A	10 (21.7%)
A/A	5 (10.9%)
VEGF rs699947, *n* (%)	
C/C	34 (74%)
C/A	-
A/A	12 (26%)
ICAM rs5498, *n* (%)	
A/A	13 (28.3%)
A/G	23 (50%)
G/G	10 (21.7%)
ICAM rs1799969, *n* (%)	
G/G	36 (78.3%)
G/A	10 (21.7%)
A/A	-
KRAS rs61764370, *n* (%)	
Wild type	12 (50%)
Mutant	12 (50%)
NRAS rs11554290, *n* (%)	
Wild type	9 (38.8%)
Mutant	15 (61.2%)
BRAF rs113488022, *n* (%)	
Wild type	20 (81.8%)
Mutant	4 (18.2%)

**Table 2:** Initial and maintenance therapeutic regimens.

Treatment Regimens	
Initial, *n* (%)	
Bevacizumab-mFOLFOX6	22 (48%)
Bevacizumab-FOLFIRI	13 (28%)
Bevacizumab-CapOX	2 (4%)
Bevacizumab-CapIRI	9 (20%)
Maintenance, *n* (%)	
Bevacizumab-mFOLFOX6	4 (9%)
Bevacizumab-FOLFIRI	5 (10%)
Bevacizumab-CapIRI	9 (20%)
Bevacizumab-De Gramont	11 (24%)
Bevacizumab-Capecitabine	3 (7%)
Bevacizumab monotherapy	14 (30%)

Bevacizumab-mFOLFOX6 (bevacizumab 5 mg/kg, oxaliplatin 85 mg/m^2^, folinic acid 400 mg/m^2^, fluorouracil 400 mg/m^2^ bolus, fluorouracil 2400 mg/m^2^ over 46 h every 2 weeks), Bevacizumab-FOLFIRI (bevacizumab 5 mg/kg, irinotecan 180 mg/m^2^, folinic acid 400 mg/m2, fluorouracil 400 mg/m^2^ bolus, fluorouracil 2400 mg/m^2^ over 46 h every 2 weeks), Bevacizumab-CapOX (bevacizumab 7.5 mg/kg on Day 1, oxaliplatin 130 mg/m^2^ on d1, capecitabine 1000 mg/m^2^/12 h Days 1–14 every 3 weeks), Bevacizumab-CapIRI (bevacizumab 7.5 mg/kg on Day 1, irenotecan 250 mg/m^2^ on d1, capecitabine 1000 mg/m^2^/12 h Days 1–14 every 3 weeks), Bevacizumab-De Gramont (bevacizumab 5 mg/kg, folinic acid 200 mg/m^2^ on Days 1,2, fluorouracil 400 mg/m^2^ bolus on Days 1,2, fluorouracil 2400 mg/m^2^ over 22 h on Days 1,2 every 2 weeks), Bevacizumab-Capecitabine (bevacizumab 7.5 mg/kg on Day 1, capecitabine 1000 mg/m^2^/12 h Days 1–14 every 3 weeks), Bevacizumab monotherapy (5 mg/kg every 2 weeks or 7.5 mg/kg every 3 weeks).

**Table 3:** Analysis of the polymorphisms for progression-free survival (PFS) and overall survival (OS).

	PFS		OS	
	Median Months (95% CI)	*p*-Value	Median Months (95% CI)	*p*-Value
VEGF rs2010963				
G/G	7.6 (4.8–10.4)	0.600	35.4 (11.6–59.2)	0.811
G/C	9.6 (5.9–13.2)		22.5 (21.5–47.6)	
C/C	8.9 (7.1–10.7)		16.9 (15–18.8)	
VEGF rs1570360		0.554		0.074
G/G	8.9 (6.9–10.9)		27.8 (20–35.6)	
G/A	12.6 (1.6–23.6)		43.2 (33.2–53.2)	
A/A	9.3 (3.7–18.9)		39.2 (12.4–65.9)	
VEGF rs699947				
C/C	8.1 (5.3–10.9)	***0.006***	16.9 (12.8–20.9)	***0.043***
C/A	N/A		N/A	
A/A	32.6 (13.2–44.9)		59.4 (33.3–70.7)	
ICAM rs5498				
A/A	8.9 (6.6–11.2)	0.700	16.9 (12.6–21.2)	0.159
A/G	8.1 (4.8–11.4)		35.4 (11.2–59.5)	
G/G	9.4 (4.6–14.2)		47.9 (26.5–85.7)	
ICAM rs1799969				
G/G	9.1 (7–11.2)	0.938	29.1 (21.4–36.9)	***0.036***
G/A	8.1 (3.5–12.7)		48.7 (34.5–62.8)	
A/A	N/A		N/A	
KRAS rs61764370				
Wild type	8.9 (3.9–13.8)	0.880	43.5 (29–57.7)	0.511
Mutant	8.1 (5.6–10.7)		20.8 (13.9–27.7)	
NRAS rs11554290				
Wild type	8.9 (6.2–11.6)	0.783	25 (19.2–30.9)	0.374
Mutant	8.1 (5.3–10.9)		33.7 (19.2–30.9)	
BRAF rs113488022				
Wild type	7.1 (6.8–7.4)	0.466	16.7 (14.3–19.1)	***0.027***
Mutant	5.6 (3.9–13.4)		6.8 (4.6–15.9)	
